# Severity of rotator cuff disorders and additional load affect fluoroscopy-based shoulder kinematics during arm abduction

**DOI:** 10.1186/s10195-024-00774-2

**Published:** 2024-06-08

**Authors:** Eleonora Croci, Hanspeter Hess, Jeremy Genter, Cornelia Baum, Balazs Krisztian Kovacs, Corina Nüesch, Daniel Baumgartner, Kate Gerber, Andreas Marc Müller, Annegret Mündermann

**Affiliations:** 1 Department of Biomedical Engineering, University of Basel, Basel, Switzerland; 2 Department of Orthopaedics and Traumatology, University Hospital Basel, Basel, Switzerland; 3 School for Biomedical and Precision Engineering, University of Bern, Bern, Switzerland; 4 IMES Institute of Mechanical Systems, Zurich University of Applied Sciences, Winterthur, Switzerland; 5 Research and Development, Shoulder and Elbow Surgery, Schulthess Klinik Zurich, Zurich, Switzerland; 6 Department of Radiology, University Hospital Basel, Basel, Switzerland; 7 Department of Spine Surgery, University Hospital Basel, Basel, Switzerland; 8 Department of Clinical Research, University of Basel, Basel, Switzerland

**Keywords:** Shoulder injury, Rotator cuff tear, Rotator cuff tendinopathy, Fluoroscopy, Handheld weight, Scaption, Scapular rotation, Glenohumeral translation

## Abstract

**Background:**

Rotator cuff disorders, whether symptomatic or asymptomatic, may result in abnormal shoulder kinematics (scapular rotation and glenohumeral translation). This study aimed to investigate the effect of rotator cuff tears on in vivo shoulder kinematics during a 30° loaded abduction test using single-plane fluoroscopy.

**Materials and methods:**

In total, 25 younger controls, 25 older controls and 25 patients with unilateral symptomatic rotator cuff tears participated in this study. Both shoulders of each participant were analysed and grouped on the basis of magnetic resonance imaging into healthy, rotator cuff tendinopathy, asymptomatic and symptomatic rotator cuff tears. All participants performed a bilateral 30° arm abduction and adduction movement in the scapular plane with handheld weights (0, 2 and 4 kg) during fluoroscopy acquisition. The range of upward–downward scapular rotation and superior–inferior glenohumeral translation were measured and analysed during abduction and adduction using a linear mixed model (loads, shoulder types) with random effects (shoulder ID).

**Results:**

Scapular rotation was greater in shoulders with rotator cuff tendinopathy and asymptomatic rotator cuff tears than in healthy shoulders. Additional load increased upward during abduction and downward during adduction scapular rotation (*P* < 0.001 in all groups but rotator cuff tendinopathy). In healthy shoulders, upward scapular rotation during 30° abduction increased from 2.3° with 0-kg load to 4.1° with 4-kg load and on shoulders with symptomatic rotator cuff tears from 3.6° with 0-kg load to 6.5° with 4-kg load. Glenohumeral translation was influenced by the handheld weights only in shoulders with rotator cuff tendinopathy (*P* ≤ 0.020). Overall, superior glenohumeral translation during 30° abduction was approximately 1.0 mm with all loads.

**Conclusions:**

The results of glenohumeral translation comparable to control but greater scapular rotations during 30° abduction in the scapular plane in rotator cuff tears indicate that the scapula compensates for rotator cuff deficiency by rotating. Further analysis of load-dependent joint stability is needed to better understand glenohumeral and scapula motion.

*Level of evidence*: Level 2.

*Trial registration*: Ethical approval was obtained from the regional ethics committee (Ethics Committee Northwest Switzerland EKNZ 2021-00182), and the study was registered at clinicaltrials.gov on 29 March 2021 (trial registration number NCT04819724, https://clinicaltrials.gov/ct2/show/NCT04819724).

**Supplementary Information:**

The online version contains supplementary material available at 10.1186/s10195-024-00774-2.

## Introduction

The primary function of the rotator cuff muscles is to facilitate shoulder movements and centre the glenohumeral joint to prevent superior glenohumeral translation during abduction [1]. Tears of rotator cuff muscles can therefore disrupt the force balance coupled with the deltoid muscle and lead to glenohumeral joint instability [2], with possible impairment of shoulder function in activities of daily living [3], resulting in lower functional scores, such as the Constant score [4, 5].

Rotator cuff tears are often associated with abnormal shoulder joint kinematics, but reported changes are inconclusive [6–9], and a comprehensive understanding of scapular and glenohumeral motion is still lacking, in part because many rotator cuff tears are asymptomatic [10]. Abnormal scapular motion has been reported in shoulders with rotator cuff tears, but no consistent pattern has been observed [11]. An increase [12–17] but also a decrease [18] or no difference [19, 20] in upward scapular rotation during arm abduction movement has been reported in shoulders with rotator cuff tears. Regarding glenohumeral translation, some studies reported a superior translation during full abduction [21, 22], while others described a superior translation only up to 90° abduction, followed by an inferior translation towards full abduction [20, 23, 24].

Some of the inconsistencies in literature regarding kinematics may arise from the different acquisition techniques, such as conventional radiographs [9, 23, 25, 26] or single/dual-plane fluoroscopy combined with three-dimensional (3D)-to-two-dimensional (2D) model-to-image registration techniques [20, 22, 24, 27–30]. In addition, shoulder kinematics may differ under quasistatic and dynamic conditions [21]. Hence, because of its capability of pulsed image acquisition, fluoroscopy is superior to conventional (isolated) radiography for the dynamic assessment of shoulder kinematics.

Shoulder rehabilitation programmes often use increasing loads to progressively challenge muscles to improve strength and function [31]. In addition, many activities of daily living involve lifting or carrying objects. However, the effect of load on shoulder kinematics has been poorly investigated. Only a few studies have examined such an effect on glenohumeral translation, and these have been in healthy shoulders, with inconsistent results [21, 23, 25, 30]. Therefore, the effect of loading on shoulder kinematics in shoulders with rotator cuff disorders remains to be investigated. The initial phase of abduction is also an important aspect of many everyday tasks. As the supraspinatus muscle is a major contributor during this phase, it is important to carefully analyse the stability of shoulders with rotator cuff disorders under different loading conditions. To date, there is a paucity of information on shoulder kinematics during initial arm abduction.

The aim of this study was to investigate the effect of rotator cuff tears on the in vivo kinematics of the glenohumeral joint during a 30° loaded and unloaded abduction test assessed by pulsed single-plane fluoroscopy and its relationship with the Constant score [4, 5]. Specifically, we aimed to determine whether scapular rotation and glenohumeral translation differ between shoulders with rotator cuff tendinopathy or symptomatic or asymptomatic rotator cuff tears and healthy controls and whether these differences increase with additional handheld weights to more closely mimic kinematics during daily activities.

## Materials and methods

### Participants

This prospective observational study (level of evidence: II) was part of a larger study on shoulder biomechanics after rotator cuff tears. The study protocol of the umbrella study has been published previously [32]. The study was approved by the regional ethics board (Ethics Committee Northwest Switzerland EKNZ 2021-00182), registered at clinicaltrials.gov (trial registration number NCT04819724) and conducted in accordance with the Declaration of Helsinki. Informed consent was obtained from all participants prior to data collection.

Older patients (45–85 years) with symptomatic unilateral rotator cuff tears and older (45–85 years) and younger (20–30 years) sex-matched control subjects were enrolled in this study between May 2021 and January 2023. Patients aged between 45 and 85 years were included in the study if a unilateral rotator cuff tear of at least the supraspinatus tendon was diagnosed and the active arm range of motion in abduction and flexion was at least 30°. Control subjects were included in the study if they were between 20 and 30 years of age (younger) or between 45 and 85 years of age (older), had an active arm range of motion of at least 90° in abduction and flexion, had no shoulder pain and had no known previous elbow or shoulder injuries. We included a group of young participants because of the reported high prevalence of asymptomatic rotator cuff tears in older adults [33, 34]. Exclusion criteria were: contraindications for magnetic resonance imaging (MRI), body mass index (BMI) greater than 35 kg/m^2^ (owing to the MRI bore size of 60 cm and the motion capture analysis of the umbrella study), inability to provide informed consent, prior operative treatments of the upper extremity (both sides), clinical history of the glenohumeral joints [only of the contralateral (non-symptomatic) side for patients, e.g. fractures and dislocations], pregnancy, neuromuscular disorders affecting upper limb movements or other pathologies influencing shoulder joint mobility.

As fully described in the published study protocol [32], 76 shoulders were required to detect a correlation of 0.37 at a significance level with 90% power.

### Testing protocol

MRIs were obtained for all shoulders using a 3-T scanner (Prisma, Siemens Healthineers, Erlangen, Germany) with dedicated shoulder and body array coils, without contrast agent. The following sequences were acquired: axial proton density turbo spin echo sequence with fat saturation, sagittal T1-weighted turbo spin echo, sagittal and coronal T2-weighted BLADE and coronal T1-weighted volumetric interpolated breath-hold examination Dixon sequence.

Single-plane fluoroscopic images (Multitom Rax, Siemens Healthineers, Erlangen, Germany) were acquired for both shoulders during a 30° loaded and unloaded arm abduction and adduction movement in the scapular plane with the extended elbow and hand in neutral position. Participants abducted both arms simultaneously to ensure an upright and stable torso and to prevent compensatory trunk movements. Shoulder abduction was performed in the scapular plane up to 30°, first without handheld weight and then with an additional handheld weight of 2 kg and 4 kg in a randomised order. Verbal commands were given to the participants during image acquisition to maintain a comparable movement speed (10°/s). Images of the right shoulder were acquired first, and then those of the left shoulder. Images were captured with a pulse rate of 3 Hz (in the first 20 participants) or 10 Hz (in the remaining 55 participants). The pulse rate was increased to avoid blurred images. A calibration sphere fixed to the fluoroscope (*Ø* = 25 mm) was placed in the scapular plane lateral and cranial of the shoulder in the field of view of the fluoroscopic images, which were taken in the scapular plane. Participants were also instructed not to actively shrug their shoulders. Finally, the Constant score [4, 5] of all shoulders was acquired.

### Data extraction

**Fig. 1:**
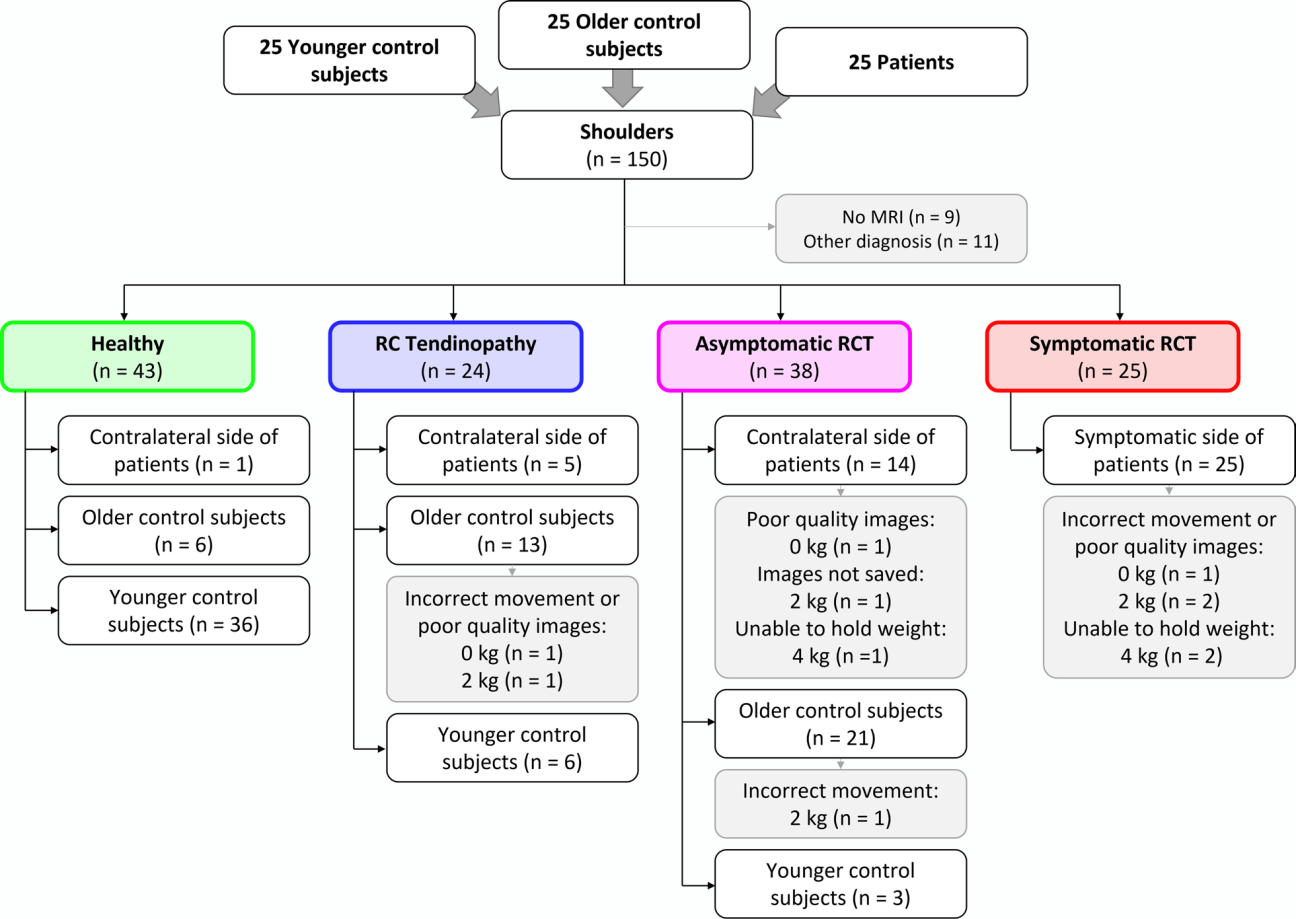
Flowchart of the study with the subdivision of shoulder types. *RC* rotator cuff, *RCT* rotator cuff tears, *MRI* magnetic resonance imaging

All shoulders were grouped according to MRI findings into the following types: healthy, with rotator cuff tendinopathy [35], with asymptomatic rotator cuff tears and with symptomatic rotator cuff tears. Shoulders with other findings not involving the rotator cuff were discarded (Fig. 1). The MRI reading was performed by an experienced radiologist in musculoskeletal imaging.

**Fig. 2:**
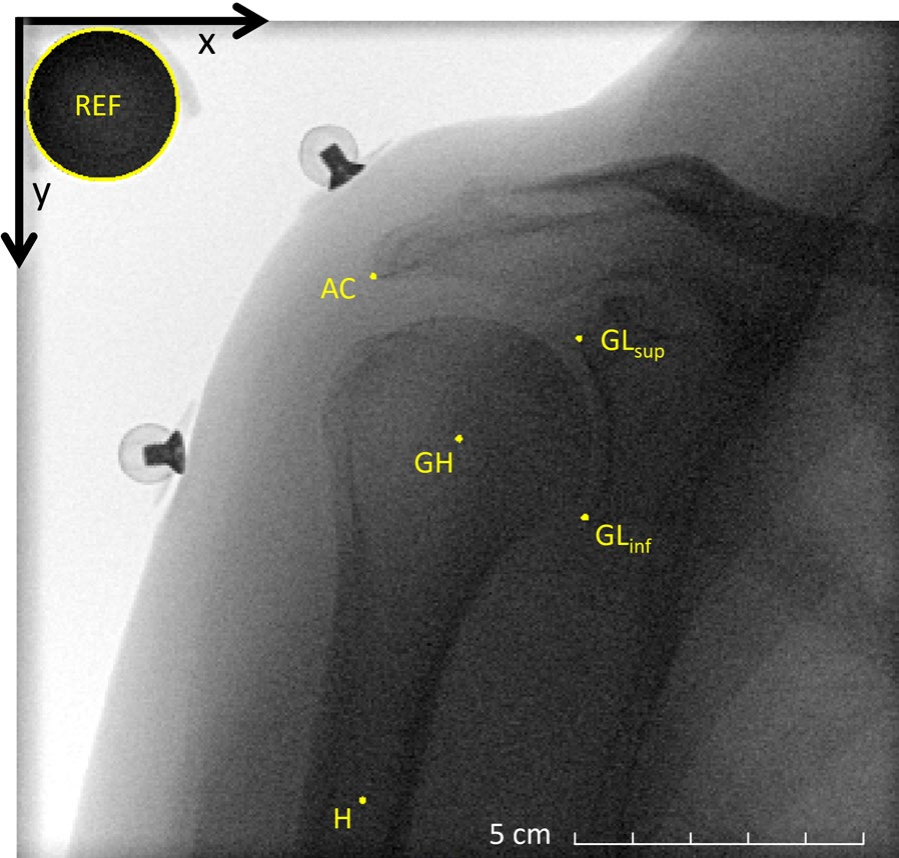
Example of a fluoroscopic image with the anatomical landmarks and the reference sphere. Image coordinates system is shown in black. *GH* glenohumeral joint centre, *H* humeral shaft midpoint, *GL*_*sup*_ superior edge of glenoid, *GL*_*inf*_ inferior edge of glenoid, *AC* most lateral point of the acromion, *REF* reference sphere

Anatomical shoulder landmarks (glenohumeral joint centre, humeral shaft midpoint, inferior and superior edges of the glenoid and most lateral point of the acromion) on each fluoroscopic image were detected with a previously published automatic algorithm on the basis of the nnU-net [36] with good accuracy (1.5 mm and 1°) [37]. All detected landmarks were manually checked again by one reader and corrected, if necessary, using 3D Slicer [38] (slicer.org). Figure 2 shows an example of a fluoroscopic image with the detected anatomical landmarks. The checked coordinates were then imported in MATLAB 2021b (The Mathworks, Natick, MA, USA) to measure upward–downward scapular rotation and superior–inferior glenohumeral translation during the entire abduction and adduction movement. Upward–downward scapular rotation was computed as rotation of the glenoid coordinate system (origin halfway between the inferior and superior edges of the glenoid, with the *y*-axis defined by these edges and the *x*-axis defined perpendicular to the *y*-axis pointing laterally) relative to the global coordinate system. Superior–inferior glenohumeral translation as the motion of the glenohumeral joint centre along the *y*-axis of the glenoid coordinate system. The abduction angle (i.e. humerothoracic angle) was measured as the angle between the line passing through the glenohumeral joint centre and the centre of the humeral shaft and the global image *y*-axis. The ranges of scapular rotation and glenohumeral translation were then measured during abduction (from initial position to maximum abduction angle) and adduction (from maximum abduction angle to end position).

### Statistical analysis

All statistical analyses were performed with RStudio (v4.2.2) [39]. Linear mixed models [40] with fixed (load and shoulder types) and random effects (shoulder ID) were applied to the scapular rotation and glenohumeral translation ranges measured during abduction and adduction. Post-hoc tests (estimated marginal means [41]) were conducted accordingly. Differences in the Constant scores were assessed with independent *t*-test with Bonferroni corrections for group analysis. In addition, Pearson’s correlation coefficient of Constant scores with scapular rotation and glenohumeral translation was investigated for the different loading conditions. The significance level was set to *P* = 0.05.

## Results

Overall, 75 participants were enrolled in this study: equally distributed between patients with unilateral symptomatic rotator cuff tear, older control subjects and younger control subjects (Table 1). All participants were able to perform the loaded and unloaded abduction movements correctly, with the exception of two patients. One patient with symptomatic unilateral rotator cuff tear could not hold the 4-kg weight with both sides, and another patient could not hold the 4-kg weight with the symptomatic side. In one case, the images of the test with 2 kg were inadvertently not saved. After MRI readings, 43 healthy shoulders, 24 shoulders with rotator cuff tendinopathy, 38 shoulders with asymptomatic rotator cuff tears, and 25 shoulders with symptomatic rotator cuff tears were included in the final analysis (Fig. 1; Table 2). As 84% of the healthy shoulders were from the younger control group, the effect of age was not examined. Overall, 20 shoulders were excluded from the analysis because of missing MRIs owing to claustrophobia (previously unknown) or other MRI findings not involving the rotator cuff (Fig. 2). Furthermore, three data points with 0 kg and four with 2 kg were excluded because of incorrect movement (elbow not extended) or poor-quality fluoroscopic images (Fig. 1).

**Table 1:** Mean and standard deviation of the descriptive data for the enrolled participants

	Patients	Older control subjects	Younger control subjects
*n*	25	25	25
Sex (male/female)	15/10	15/10	15/10
Age (years)	64.3 ± 10.2	55.4 ± 8.2	26.1 ± 2.3
Height (cm)	172 ± 10	174 ± 9	177 ± 9
Body mass (kg)	78.4 ± 17.3	76.5 ± 13.0	71.6 ± 12.9
BMI (kg/m^2^)	26.5 ± 5.0	25.2 ± 4.6	22.7 ± 3.0

The participants were later re-grouped according to the findings on magnetic resonance images

*BMI* body mass index

**Table 2:** Severity of rotator cuff tears of the symptomatic and asymptomatic shoulders

	Asymptomatic shoulders	Symptomatic shoulders
*n*	38	25
Partial tears	81.6%	48%
Single full-thickness tear	2.6%	12%
Two or three tendons (only one full-thickness tear)	13.2%	32%
Massive tears (two or more full-thickness tears)	2.6%	8%

### Scapular rotation

**Fig. 3:**
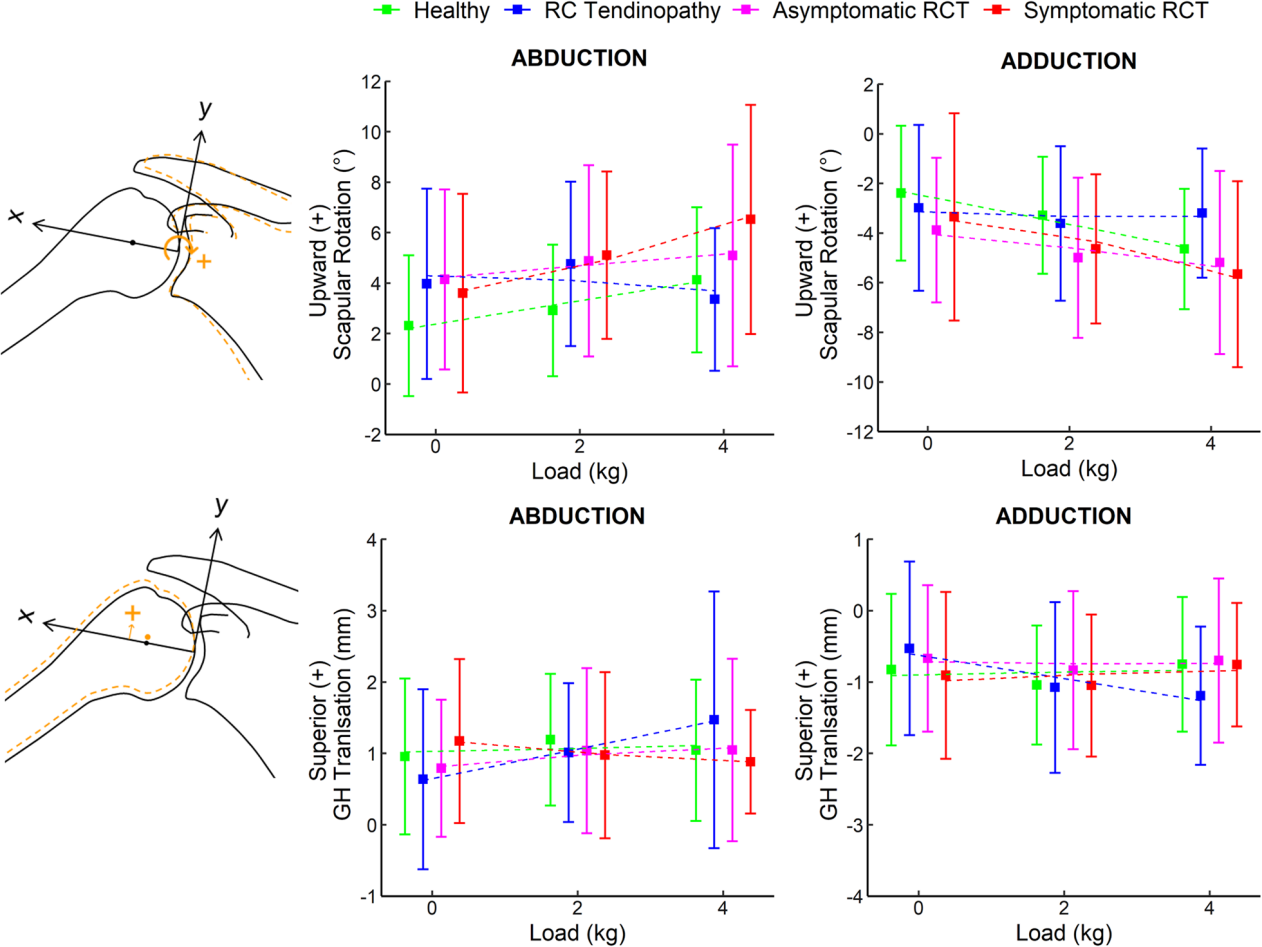
Mean and standard deviation of the scapular rotations (above) and glenohumeral (GH) translations (below) during abduction and adduction for the four shoulder types with regression lines (dashed). In the sketches on the left, scapular rotation (above) and glenohumeral translation (below) are shown with the axes of the glenoid coordinate system (*x*, *y*). *RC* rotator cuff, *RCT* rotator cuff tears

At the initial, maximum abduction and end positions, all shoulder types had similar extrinsic glenoid inclination [42] (Supplementary Fig. S1). The range of upward scapular rotation during 30° abduction was smallest in healthy shoulders and greatest in shoulders with symptomatic rotator cuff (Fig. 3; see Supplementary Table S1 for mean and standard deviation). With increasing load, the load-induced increase in upward scapular rotation increased (Fig. 3; Table 3). In addition, shoulders with rotator cuff tendinopathy and shoulders with asymptomatic rotator cuff tears differed significantly in scapular rotation (greater upward rotation) compared with healthy shoulders (Table 3). An interaction effect between load and rotator cuff tendinopathy was observed, with a smaller increase in upward scapular rotation with increasing load in shoulders with rotator cuff tendinopathy compared with healthy shoulders (Table 3). Post-hoc tests of the linear mixed model for shoulder types showed that upward scapular rotation differed significantly between shoulders with asymptomatic rotator cuff tears and healthy shoulders at 0-kg load (*P* = 0.048), between shoulders with symptomatic rotator cuff tears and healthy shoulders at 2-kg and 4-kg loads (*P* = 0.025 and *P* = 0.006, respectively) and between shoulders with rotator cuff tendinopathy and shoulders with symptomatic rotator cuff tears at 4-kg load (*P* = 0.006). No other significant differences were observed between shoulder types at any load (*P* > 0.05). Post-hoc tests for load effect revealed significant differences in scapular rotation between all load comparisons in healthy shoulders (*P* < 0.001) and in shoulders with symptomatic rotator cuff tears (*P* < 0.001).

**Table 3:** Effects of load and shoulder status on scapular rotation and glenohumeral translation during abduction and adduction

Predictors	Scapular rotation				Glenohumeral translation			
	Abduction		Adduction		Abduction		Adduction	
	Estimates (CI)	*P*-value	Estimates (CI)	*P*-value	Estimates (CI)	*P*-value	Estimates (CI)	*P*-value
Intercept	2.21 (1.20 to 3.22)	** < 0.001**	−2.31 (−3.20 to −1.42)	** < 0.001**	1.02 (0.70 to 1.34)	** < 0.001**	−0.91 (−1.21 to −0.61)	** < 0.001**
Load	0.45 (0.21 to 0.70)	** < 0.001**	−0.56 (−0.79 to −0.34)	** < 0.001**	0.02 (−0.07 to 0.12)	0.642	0.02 (−0.06 to 0.10)	0.645
RC tendinopathy	1.95 (0.24 to 3.65)	**0.026**	−0.68 (−2.19 to 0.82)	0.373	−0.40 (−0.93 to 0.14)	0.146	0.31 (−0.19 to 0.81)	0.224
Asymptomatic RCT	1.96 (0.48 to 3.45)	**0.009**	−1.77 (−3.09 to −0.46)	**0.008**	−0.21 (−0.68 to 0.26)	0.372	0.20 (−0.24 to 0.64)	0.368
Symptomatic RCT	1.43 (−0.25 to 3.11)	0.094	−1.12 (−2.61 to 0.36)	0.139	0.14 (−0.39 to 0.67)	0.598	−0.08 (−0.57 to 0.42)	0.759
Load × RC tendinopathy	−0.58 (−0.98 to −0.17)	**0.005**	0.48 (0.10–0.86)	**0.014**	0.19 (0.03 to 0.34)	**0.020**	−0.18 (−0.32 to −0.05)	**0.007**
Load × asymptomatic RCT	−0.16 (−0.52 to 0.19)	0.379	0.24 (−0.09 to 0.58)	0.155	0.05 (−0.09 to 0.19)	0.481	−0.03 (−0.15 to 0.09)	0.617
Load × symptomatic RCT	0.36 (−0.05 to 0.77)	0.083	−0.10 (−0.48 to 0.29)	0.607	−0.11 (−0.26 to 0.05)	0.187	0.03 (−0.10 to 0.16)	0.661

Bold values indicate significant differences (*P* < 0.05)

*RC* rotator cuff, *RCT *rotator cuff tear, *CI *confidence interval

Similarly, in adduction, healthy shoulders had the smallest amount and shoulders with symptomatic rotator cuff tears had the largest amount of downward scapular rotation (Fig. 3). A load-induced increase in downward scapular rotation was observed with greater additional load (Table 3). In addition, shoulders with asymptomatic rotator cuff tears had significantly greater downward scapular rotation than healthy shoulders (Table 3). A significant interaction between load and shoulders with rotator cuff tendinopathy was found, where the load-induced increase in downward scapular rotation was attenuated with increasing load in shoulders with rotator cuff tendinopathy compared with healthy shoulders (Table 3). Post-hoc tests revealed significant differences in downward scapular rotation between shoulders with asymptomatic rotator cuff tears and healthy shoulders at 0-kg load (*P* = 0.041). Furthermore, a significant increase in downward scapular rotation was found between shoulders with rotator cuff tendinopathy and shoulders with rotator cuff tears at 4-kg load (asymptomatic: *P* = 0.049, symptomatic: *P* = 0.009). No other significant differences were found between shoulder types at any load (*P* > 0.05). Additionally, the 4-kg load increment and all 2-kg load increments led to increased downward scapular rotation in healthy shoulders (*P* < 0.001) and in shoulders with rotator cuff tears (asymptomatic: *P* = 0.031, symptomatic: *P* < 0.001).

In both models for abduction and adduction, the fixed effects (marginal *R*^2^) explained less variance than the random effects (conditional *R*^2^; Table 4). Results (*P*-values) of the linear mixed models with the different shoulder types as reference can be found in Supplementary Table S2.

**Table 4:** Random effects of the linear mixed models for scapular rotations and glenohumeral translations

	Scapular rotation		Glenohumeral translation	
	Abduction	Adduction	Abduction	Adduction
Intraclass correlation	0.580	0.525	0.389	0.483
Marginal *R*^2^	0.096	0.101	0.024	0.019
Conditional *R*^2^	0.620	0.574	0.404	0.493

### Glenohumeral translation

At the initial, maximum abduction and end positions, all shoulder types had a similar positioning of the humeral head relative to the glenoid with the humeral head slightly inferior to the centre of the glenoid at the initial position (Supplementary Fig. S2). Glenohumeral translation was not affected by the different handheld weights during 30° abduction or adduction in any of the shoulder types (Fig. 3; Table 3). Only one significant interaction effect between load and shoulders with rotator cuff tendinopathy was found in both abduction and adduction (Table 3), where glenohumeral translation increased with increasing load superiorly (abduction) or inferiorly (adduction). Post-hoc tests revealed a significant difference between all load comparisons only for shoulders with rotator cuff tendinopathy (abduction: *P* = 0.004, adduction: *P* = 0.007). In both models for abduction and adduction, the fixed effects (marginal *R*^2^) explained less variance than the random effects (conditional *R*^2^; Table 4).

### Constant score

Mean ± standard deviation of Constant scores were 88.2 ± 2.6 in healthy shoulders, 85.2 ± 6.1 in shoulders with rotator cuff tendinopathy, 84.0 ± 5.2 in shoulders with asymptomatic rotator cuff tears and 74.0 ± 10.3 in shoulders with symptomatic rotator cuff tears. The Constant scores in shoulders with symptomatic rotator cuff tears differed from all other shoulder types (*P* < 0.001). Shoulders with symptomatic rotator cuff tears presented some range of motion deficit and pain with respect to the other shoulder types. The remaining differences were related to scores achieved in the strength component of the Constant score. Significant differences were also found between shoulders with asymptomatic rotator cuff tears and healthy shoulders (*P* < 0.001). Constant scores did not differ between shoulders with asymptomatic rotator cuff tears and shoulders with rotator cuff tendinopathy (*P* = 1.000) or between shoulders with rotator cuff tendinopathy and healthy shoulders (*P* = 0.187).

### Association between shoulder kinematics and constant scores

A low correlation was found between the Constant score and scapular rotation for the 2-kg load during abduction (*R* = 0.230, *P* = 0.010). No other relevant correlations were detected between the Constant scores and scapular rotation (−0.127 ≤ *R* ≤ 0.120, *P* > 0.05) or glenohumeral translation (−0.123 ≤ *R* ≤ 0.143, *P* > 0.05) for the other loading conditions during abduction or adduction.

## Discussion

In this study, scapular and glenohumeral kinematics of 130 shoulders with and without rotator cuff disorders were examined during a bilateral 30° loaded and unloaded abduction test in the scapular plane. Most of the incidental findings on MRI occurred in participants over 45 years of age. Most of the asymptomatic shoulders had partial tears, while more than half of the symptomatic shoulders had at least a full-thickness supraspinatus tear (kinematics were not significantly different; Supplementary Table S3). Using single-plane fluoroscopy, we were able to evaluate both upward–downward scapular rotation and superior–inferior glenohumeral translation during a 30° loaded (up to 4 kg) and unloaded abduction test in shoulders with rotator cuff disorders and in healthy shoulders. Load-induced changes in scapular rotation were assessed in healthy shoulders and in shoulders with rotator cuff tears, while glenohumeral translation was unaffected by the additional handheld weights. Overall, the kinematic changes that occurred during 30° abduction, such as upward scapular rotation and superior glenohumeral translation, were mainly restored during 30° adduction with downward scapula rotation and inferior glenohumeral translation.

### Shoulder kinematics in the presence of rotator cuff disorders

In the unloaded condition, upward scapular rotation during the 30° abduction movement increased to 2.3° in healthy shoulders and to 3.6° in shoulders with symptomatic rotator cuff tears. A similar increase in upward scapular rotation during abduction was reported in a suprascapular nerve block study [43]. In addition, Miura et al. [13] reported – albeit with slightly smaller values – an average increase in upward scapular rotation of 0.1° in healthy shoulders and of 3.3° in shoulders with massive rotator cuff tears. Their study used an electromagnetic tracking system, which might underestimate scapular motion owing to skin motion artefacts. In our study, kinematic differences were observed in scapular motion between pathologic and healthy shoulders, with shoulders with symptomatic rotator cuff tears having the greatest upward scapular rotation and healthy shoulders having the least upward scapular rotation during the 30° abduction movement. Some differences in scapular rotation were found between shoulders with rotator cuff tears (asymptomatic or symptomatic) and healthy shoulders, and also between shoulders with symptomatic rotator cuff tears and shoulders with rotator cuff tendinopathy at some loads. However, no major differences were found between shoulders with symptomatic and asymptomatic rotator cuff tears up to 30° of abduction. The presence of symptoms may thus be related to different compensatory muscle activities. Indeed, in the same umbrella study [32, 44], higher muscle activities in the posterior deltoid and pectoralis major were found between symptomatic and asymptomatic rotator cuff tears.

The finding of greater upward scapular rotation after rotator cuff tears is consistent with other kinematics studies [12–17] and is further supported by electromyography studies [45, 46], which have observed increased axioscapular (upper trapezius, serratus anterior) muscle activity as an indication of increased upward scapular rotation. Thus, the increased scapular rotation in shoulders with rotator cuff disorders may reflect a compensatory mechanism for the deficient rotator cuff muscles to counteract the decreased abduction torque.

Regarding glenohumeral translation, we observed a superior glenohumeral translation of 1.0 and 1.2 mm during the 30° abduction movement without additional weight in healthy shoulders and in shoulders with symptomatic rotator cuff tears, respectively. Similar values were reported by Kozono et al. [24] for 30° of abduction in the scapular plane, and hence, these small glenohumeral translations may simply be physiological. Although there were no significant differences between shoulder types in our study, shoulders with symptomatic rotator cuff tears may tend to have slightly larger superior glenohumeral translations because of the missing centralising action of the rotator cuff and greater activity in the deltoid muscle, as also supported by Kijima et al. [20]. Moreover, rotator cuff tears involving the infraspinatus tendon are likely to be associated with larger glenohumeral translations [47].

### Load-induced kinematic changes

We observed a load-induced increase in scapular rotation in healthy shoulders and in shoulders with rotator cuff tears. Glenohumeral translation changed with additional weight neither in healthy shoulders, as reported also by Nishinaka et al. [30], nor in shoulders with rotator cuff tears during 30° abduction. Therefore, in this cohort, shoulders with rotator cuff tears and healthy shoulders appear to behave similarly under load, with upward scapular rotation increasing during 30° abduction so as not to alter glenohumeral joint stability. Thus, maintaining regular glenohumeral motion may be more important than maintaining regular scapular motion during abduction initiation. However, this behaviour may change at larger abduction angles, when the surrounding muscles have reached their maximum level of activation, possibly leading to greater glenohumeral translation. From a muscular point of view, it is possible that the additional load results in increased activity of the deltoid and the intact rotator cuff muscles, which are then counterbalanced by increased activity of the axiohumeral muscles (pectoralis major, latissimus dorsi), so that the axioscapular muscles (trapezius, serratus anterior) also become more active to perform the movement [44].

Shoulders with rotator cuff tendinopathy showed a different trend with additional loads during 30° of abduction, as a load-induced increase in superior (abduction) or inferior (adduction) glenohumeral translation was observed, while scapular rotation remained constant, possibly as a result of the electromyographic changes observed in the larger umbrella study, such as increased deltoid muscle activity and almost unchanged latissimus dorsi muscle activity with additional load [44]. In particular, the smaller load-induced increase in the latissimus dorsi muscle activity in shoulders with rotator cuff tendinopathy compared with the other shoulders may be related to greater glenohumeral translation. Hence the superiorly directed force of the deltoid (and supraspinatus) muscles is less counterbalanced by the adductor activity of the latissimus dorsi. However, the load-induced changes were small with a mean absolute change of 0.9 mm during 30° abduction with 4-kg load compared with 0-kg load. Depending on the load and the extent of the rotator cuff pathology, the level of compensation required may be greater or smaller, resulting in different scapular rotation.

### Clinical relevance

The abduction test using single-plane fluoroscopy, as used in our study, could be implemented in clinical practice owing to the efficient image acquisition and processing to obtain accurate shoulder kinematics, which may help in deciding the treatment strategy. This method allows obtaining accurate glenohumeral kinematics and assessing compensatory strategies under different loading conditions that could be addressed with therapy [48] without exposing patients to excessive radiation. The absence of a strong association between the results of the abduction test and the Constant score suggests that these two tools may assess different aspects of shoulder functionality with rotator cuff tears. Overall, lower Constant scores are to be expected in symptomatic rotator cuff tears, but the abduction test might perceive subtle variations in the joint function. A greater instability might progressively wear down the joint and lead to secondary damage, such as osteoarthritis [49]. An accurate kinematics investigation may thus contribute to an in-depth assessment of the shoulder joint. Although only a 30° abduction–adduction movement was analysed in this study, greater upward scapular rotation was found in shoulders with rotator cuff disorders, and additional load also increased upward scapular rotation during 30° abduction. This suggests that the scapulohumeral rhythm is likely to decrease in the presence of rotator cuff disorders [12] or with load, which may be important to consider in clinical decision-making and physiotherapy training.

### Strengths and limitations

A major strength of our study is the assessment of shoulder kinematics on fluoroscopic images combined with MRI findings to better investigate and objectively quantify potential compensatory mechanisms in shoulders with rotator cuff disorders, as these may be asymptomatic. As a result, the types of tears in shoulders with asymptomatic and symptomatic rotator cuff tears were not homogeneous, as all shoulders with asymptomatic rotator cuff tears were incidental findings. Because of this regrouping, we were not able to account for side dependency in the analyses between both shoulders (e.g. affected side of patients and contralateral side). In addition, we were unable to assess the effect of age on shoulder kinematics owing to the large number of incidental findings, resulting in few healthy shoulders within the older group. The kinematic results show some variability to which several aspects may have contributed. In this study, we acquired images with single-plane fluoroscopy, and scapular rotation and glenohumeral translation were calculated using anatomical landmarks on the scapula and humerus. Kinematic accuracy would presumably have been higher with dual-plane fluoroscopy and the use of a 3D-to-2D model-to-image registration technique to track the scapula and humerus. However, the availability of dual-plane fluoroscopy in a clinical setting is limited, and bone models derived from computed tomography are required, which together would expose patients to high doses of radiation [50]. Single-plane fluoroscopy provides sufficient accuracy for in-plane motion [51], as in our case with image acquisition and arm movement in the scapular plane, and patients are exposed to low radiation doses. Because of the single-plane fluoroscopy, scapular rotation and glenohumeral translation could only be measured in one direction (upward–downward and superior–inferior, respectively).

Some of the variability in the data might be due to slightly different movement velocity, although verbal instructions were given during testing, or to small movement deviation from the scapular plane, since movement was not restricted. The extent and heterogeneity of the rotator cuff disorders among participants might also be responsible for some of the variability in scapular and glenohumeral kinematics. However, more study participants would be needed to specifically analyse a particular type of rotator cuff tear and its severity. Because of the small number of massive tears in this study, results of this study might not be generalizable to such a group. In addition, the handheld weights were not selected according to the individual strength capacity of each participant. This may have resulted in some variability in glenohumeral kinematics, as the relative effort to perform the test may have differed between participants, resulting in different levels of shoulder muscle activation. Nonetheless, the results of this study are clinically relevant. This 30° arm abduction and adduction movement with additional weights allowed the effect of load on shoulder kinematics to be studied in shoulders with rotator cuff disorders and in healthy shoulders. This test provides objective measures of shoulder instability, and relevant biomechanical changes were observed.

## Conclusions

This 30° abduction test with and without additional weights and the use of single-plane fluoroscopy allowed the shoulder kinematics to be objectively assessed by exposing the patient to a low dose of radiation. Shoulder kinematics are load dependent, and this needs to be considered in shoulder rehabilitation programmes, especially in the presence of rotator cuff tears. The results of comparable glenohumeral translation to the control group (at least during 30° abduction) but greater upward scapular rotations in shoulders with rotator cuff tears, which increase with additional load, suggest that the scapula compensates for rotator cuff deficiency by rotating. Further analysis of load-dependent joint stability is needed to better understand glenohumeral and scapular motion during more demanding activities of daily living.

### Supplementary Information

Supplementary Material 1.

## Data Availability

All data generated or analysed during this study are included in this published article and its supplementary information files.
