# Supplementary material for: Severity of rotator cuff disorders and additional load affect fluoroscopy-based shoulder kinematics during arm abduction
Source: J Orthop Traumatol. 2024 Jun 8;25:30. doi: 10.1186/s10195-024-00774-2 (PMC11162404; doi:10.1186/s10195-024-00774-2)
Supplement: Supplementary file 1 — Supplementary Material 1. [file 10195_2024_774_MOESM1_ESM.pdf]

Supplementary Files

Supplementary Figure 1: Mean and standard deviation of the extrinsic glenoid inclination (Glh) for the different shoulder types and loading conditions at the start position, max. abduction and end position of the 30° abduction test in the scapular plane. No statistical significance differences were found between any comparison at the start position, max. abduction or end position.

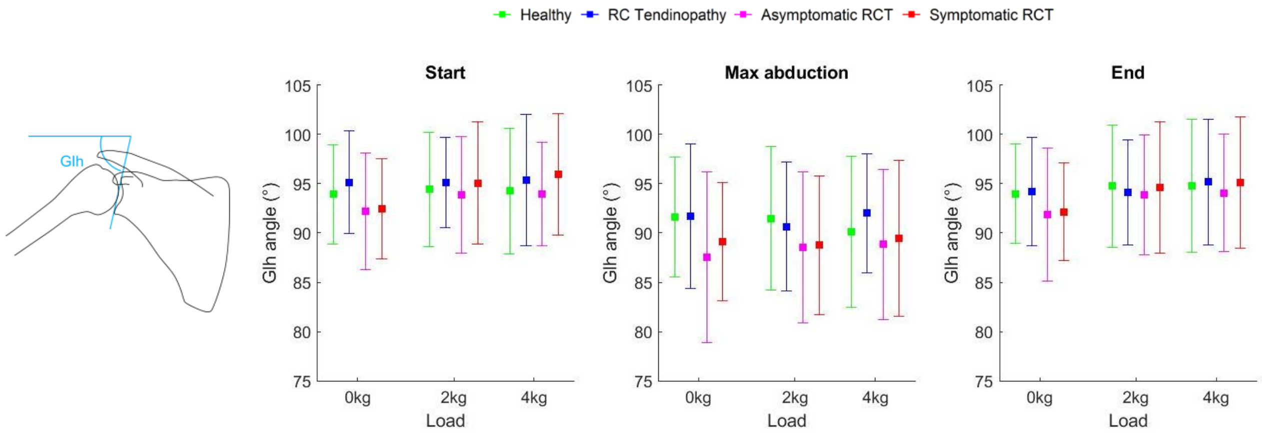

Supplementary Figure 2: Mean and standard deviation of the humeral head position for the different shoulder types and loading conditions at the start position, max. abduction and end position of the 30° abduction test in the scapular plane. No statistical significance differences were found between any comparison at the start position, max. abduction or end position.

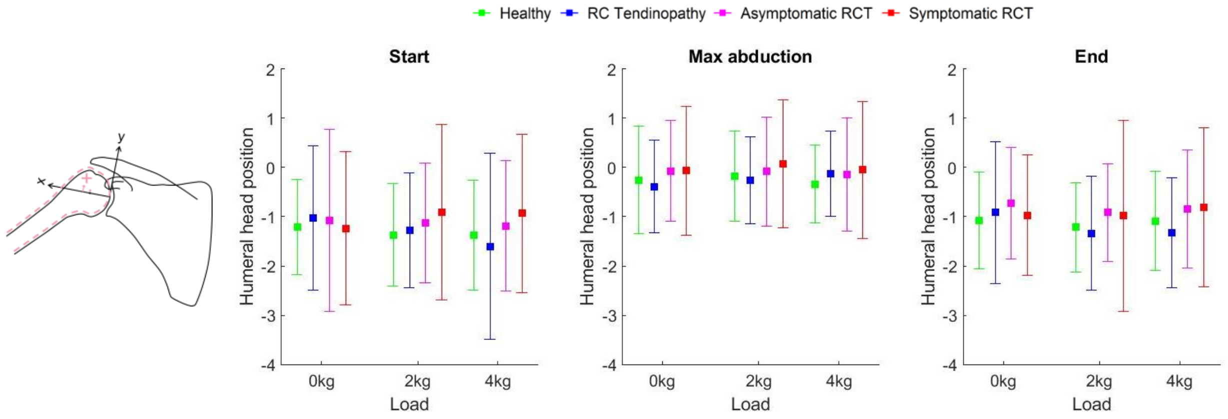

Supplementary Table 1: Mean and standard deviation of upward scapula rotation and glenohumeral translation for the different shoulder types during 30 ° abduction and adduction in the scapular plane.

	**Upward (+) scapular rotation** Mean (standard deviation) (°)			**Superior (+) glenohumeral translation** Mean (standard deviation) (mm)		
	0kg	*2kg*	*4kg*	*0kg*	*2kg*	*4kg*
**Abduction**						
Healthy	2.3 (2.8)	2.9 (2.6)	4.1 (2.9)	1.0 (1.1)	1.2 (0.9)	1.0 (1.0)
RC Tendinopathy	4.0 (3.8)	4.8 (3.3)	3.4 (2.8)	0.6 (1.3)	1.0 (1.0)	1.5 (1.8)
Asymptomatic RCT	4.1 (3.6)	4.9 (3.8)	5.1 (4.4)	0.8 (1.0)	1.0 (1.2)	1.1 (1.3)
Symptomatic RCT	3.6 (3.9)	5.1 (3.3)	6.5 (4.6)	1.2 (1.2)	1.0 (1.2)	0.9 (0.7)
**Adduction**						
Healthy	-2.4 (2.8)	-3.3 (2.4)	-4.6 (2.4)	-0.8 (1.1)	-1.0 (0.8)	-0.8 (0.9)
RC Tendinopathy	-3.0 (3.3)	-3.6 (3.1)	-3.2 (2.6)	-0.5 (1.2)	-1.1 (1.2)	-1.2 (1.0)
Asymptomatic RCT	-3.9 (2.9)	-5.0 (3.2)	-5.2 (3.7)	-0.7 (1.0)	-0.8 (1.1)	-0.7 (1.2)
Symptomatic RCT	-3.4 (4.2)	-4.6 (3.0)	-5.7 (3.8)	-0.9 (1.2)	-1.1 (1.0)	-0.8 (0.9)

RC—Rotator cuff; RCT—Rotator cuff tear.

Supplementary Table 2: P-values for the effects of load and shoulder types of the linear mixed models on the scapula rotation and glenohumeral translation with change of reference.

**Predictors**		**Upward Scapular Rotation**		**Superior Glenohumeral Translation**	
		Abduction	*Adduction*	*Abduction*	*Adduction*
(Intercept)					
	Ref: Healthy	**<0.001**	**<0.001**	**<0.001**	**<0.001**
	Ref: RC Tendinopathy	**<0.001**	**<0.001**	**0.005**	**0.003**
	Ref: Asymptomatic RCT	**<0.001**	**<0.001**	**<0.001**	**<0.001**
	Ref: Symptomatic RCT	**<0.001**	**<0.001**	**<0.001**	**<0.001**
Load					
	Ref: Healthy	**<0.001**	**<0.001**	0.642	0.645
	Ref: RC Tendinopathy	0.459	0.589	**0.001**	**0.002**
	Ref: Asymptomatic RCT	**0.028**	**0.012**	0.167	0.798
	Ref: Symptomatic RCT	**<0.001**	**<0.001**	0.194	0.375
Healthy					
	Ref: Healthy	-	**-**	**-**	-
	Ref: RC Tendinopathy	**0.025**	0.373	0.146	0.224
	Ref: Asymptomatic RCT	**0.010**	**0.008**	0.372	0.368
	Ref: Symptomatic RCT	0.094	0.139	0.598	0.759
RC Tendinopathy					
	Ref: Healthy	**0.026**	0.373	0.146	0.224
	Ref: RC Tendinopathy	-	-	**-**	-
	Ref: Asymptomatic RCT	0.985	0.167	0.514	0.677
	Ref: Symptomatic RCT	0.599	0.610	0.079	0.176
Asymptomatic RCT					
	Ref: Healthy	**0.009**	**0.008**	0.372	0.368
	Ref: RC Tendinopathy	0.985	0.167	0.514	0.677
	Ref: Asymptomatic RCT	-	-	**-**	-
	Ref: Symptomatic RCT	0.094	0.404	0.199	0.282
Symptomatic RCT					
	Ref: Healthy	0.094	0.139	0.598	0.759
	Ref: RC Tendinopathy	0.599	0.610	0.079	0.176
	Ref: Asymptomatic RCT	0.546	0.404	0.199	0.282
	Ref: Symptomatic RCT	-	**-**	**-**	-
Load x Healthy					
	Ref: Healthy	-	**-**	**-**	-
	Ref: RC Tendinopathy	**0.005**	**0.014**	**0.020**	**0.007**
	Ref: Asymptomatic RCT	0.379	0.155	0.481	0.617
	Ref: Symptomatic RCT	0.083	0.607	0.187	0.661
Load x RC Tendinopathy					
	Ref: Healthy	**0.005**	**0.014**	**0.020**	**0.007**
	Ref: RC Tendinopathy	**-**	-	**-**	-
	Ref: Asymptomatic RCT	0.051	0.243	0.097	**0.028**
	Ref: Symptomatic RCT	**<0.001**	**0.010**	**0.001**	**0.006**
Load x Asymptomatic RCT					
	Ref: Healthy	0.379	0.155	0.481	0.617
	Ref: RC Tendinopathy	0.051	0.243	0.097	**0.028**
	Ref: Asymptomatic RCT	-	-	**-**	-
	Ref: Symptomatic RCT	**0.016**	0.090	0.061	0.395
Load x Symptomatic RCT					
	Ref: Healthy	0.083	0.607	0.187	0.661
	Ref: RC Tendinopathy	**<0.001**	**0.010**	**0.001**	**0.006**
	Ref: Asymptomatic RCT	**0.016**	0.090	0.061	0.395
	Ref: Symptomatic RCT	-	-	**-**	-

Bold values indicate significant differences ($P < 0.05$). RC—Rotator cuff; RCT—Rotator cuff tear; Ref—Reference.

Supplementary Table 3: Mean and standard deviation of upward scapula rotation and glenohumeral translation for asymptomatic and symptomatic partial and full-thickness tears during 30 ° abduction and adduction in the scapular plane. No group comparisons (independent t-test) were statistically significant (P > 0.05).

	**Upward (+) scapular rotation** Mean (standard deviation) (°)			**Superior (+) glenohumeral translation** Mean (standard deviation) (mm)		
	0kg	*2kg*	*4kg*	*0kg*	*2kg*	*4kg*
**Abduction**						
Asymptomatic partial tears	4.1 (3.2)	5.1 (3.8)	5.6 (4.3)	0.8 (0.8)	1.0 (0.9)	1.0 (1.2)
Asymptomatic full-thickness tears	4.5 (5.4)	3.8 (3.8)	3.0 (4.3)	0.6 (1.6)	1.4 (2.0)	1.4 (1.8)
Symptomatic partial tears	2.0 (3.9)	4.0 (2.3)	5.2 (2.7)	1.0 (1.4)	1.3 (1.0)	0.9 (0.8)
Symptomatic full-thickness tears	5.1 (3.7)	6.4 (3.9)	7.9 (5.9)	1.4 (1.0)	0.8 (1.4)	0.8 (0.8)
**Adduction**						
Asymptomatic partial tears	-3.7 (2.6)	-5.3 (3.2)	-5.2 (3.9)	-0.7 (1.0)	-0.8 (1.0)	-0.7 (1.0)
Asymptomatic full-thickness tears	-4.6 (4.5)	-3.7 (3.4)	-5.1 (3.2)	-0.5 (1.4)	-1.0 (1.7)	-0.5 (1.4)
Symptomatic partial tears	-5.2 (3.9)	-2.8 (3.7)	-4.9 (3.3)	-0.7 (1.1)	-0.9 (1.0)	-0.7 (0.7)
Symptomatic full-thickness tears	-4.1 (4.8)	-4.6 (3.1)	-5.9 (4.6)	-1.0 (1.3)	-1.2 (1.1)	-0.8 (1.1)
